# Sharp Instruments

**Published:** 1904-06

**Authors:** K. Moyer

**Affiliations:** Little Falls, Minn.


### SHARP INSTRUMENTS.

By K. Moyer, Little Falls, Minn.

To always have sharp instruments an excellent rule to adopt is to never return an instrument to the case until it has been re-sharpened, and if followed a very few strokes on the stone brings the edge to almost a razor keenness, which makes its use a pleasure. This work can be done with an Arkansas stone on the lathe-head, but is not liable to be done as well, or rather is overdone by using up more of the instrument than is necessary at each resharpening.—*Extract Cosmos.*

